# Clinical Approaches and Emerging Therapeutic Horizons in Primary Hyperoxaluria

**DOI:** 10.3390/jcm15030940

**Published:** 2026-01-23

**Authors:** Ruth Martínez-Galindo, María Campuzano-Pérez, Afroditi Konstantouli, María Del Pilar Aguilar-Ramírez, Juan Antonio Mainez Rodríguez, Pablo Abad-López, Amir Shabaka, Ramón Cansino

**Affiliations:** 1 Nephrology Department, Hospital Universitario La Paz, 28046 Madrid, Spain; ruth.m.galindo@salud.madrid.org (R.M.-G.); maria.konstantouli@salud.madrid.org (A.K.); amir.shabaka@salud.madrid.org (A.S.); 2 Urology Department, Hospital Universitario La Paz, 28046 Madrid, Spain; maria.campuzano@salud.madrid.org (M.C.-P.); mariadelpilar.aguilar@salud.madrid.org (M.D.P.A.-R.); juan.mainez@salud.madrid.org (J.A.M.R.); pablo.abad@salud.madrid.org (P.A.-L.)

**Keywords:** primary hyperoxaluria, oxalate metabolism, RNA interference therapy, pyridoxine, kidney stones

## Abstract

Primary hyperoxalurias (PHs) are rare autosomal recessive disorders characterized by overproduction of oxalate, a metabolic end product that readily forms calcium oxalate crystals. Excess hepatic oxalate leads to recurrent kidney stones, nephrocalcinosis, and progressive renal injury, often culminating in end-stage kidney disease (ESKD). Once renal clearance declines, systemic oxalate accumulation can cause multisystem deposition. PH encompasses three types—PH1, PH2, and PH3—caused by deficiencies in the hepatic enzymes AGT, GRHPR, and HOGA1, respectively, resulting in accumulation of glyoxylate and subsequent oxalate overproduction. Clinical presentation varies from infantile oxalosis to adult-onset recurrent nephrolithiasis, with PH1 generally being the most severe. Diagnosis relies on urinary oxalate measurements, plasma oxalate in advanced chronic kidney disease, urinary metabolite profiling, imaging, and genetic testing. Management includes hyperhydration, citrate supplementation, pyridoxine for responsive PH1 patients, dialysis and transplantation when required, while RNA interference therapies targeting glycolate oxidase or LDHA have demonstrated substantial biochemical efficacy in PH1 and represent promising emerging therapeutic options, although long-term clinical outcome data remain limited and broader applicability to other PH types is still under investigation. Future strategies focus on modulating intestinal oxalate absorption, gut microbiome therapies, oxalate-degrading enzymes, and novel gene-editing approaches. Early diagnosis and individualized management are critical to prevent kidney injury and systemic oxalosis. In this review, we summarize the genetic, biochemical, and clinical features of PH and discuss current and emerging therapeutic strategies.

## 1. Introduction

Primary hyperoxaluria (PH) is a rare autosomal recessive disorders marked by the overproduction of oxalate, a metabolic end product that readily forms calcium oxalate (CaOx) crystals. In affected individuals, the liver produces excessive oxalate, which is excreted in the urine and predisposes to crystal deposition within the renal tubules. This process promotes recurrent kidney stones and nephrocalcinosis, ultimately leading to progressive renal injury and, in many cases, end-stage kidney disease (ESKD) [1].

ESKD in PH arises from a combination of tubular toxicity induced by oxalate, deposition of CaOx within the tubules and interstitium, and obstruction caused by stones [2]. Once glomerular filtration rate (GFR) falls below approximately 30–45 mL/min/1.73 m^2^, renal clearance of oxalate becomes insufficient, triggering a feedback loop in which plasma oxalate levels rise. Elevated systemic oxalate can deposit in multiple organs—including bone, retina, skin, and heart—resulting in systemic oxalosis, a potentially life-threatening complication [3,4].

Despite its severe consequences, PH is often diagnosed late, frequently after years of recurrent nephrolithiasis or once ESKD has developed. Retrospective analyses, however, suggest that symptoms may appear during early childhood. Recognition of recurrent stones or CaOx deposition in the kidneys should raise clinical suspicion for PH in both pediatric and adult patients [5]. In pediatric populations, PH is frequently underrecognized, with diagnosis often delayed due to misclassification as idiopathic nephrolithiasis or recurrent urinary tract infection. Delayed diagnosis increases the risk of irreversible renal damage and systemic oxalosis. Recent therapeutic advances, particularly RNA interference–based therapies, have the potential to alter the historical natural history of the disease by enabling earlier metabolic control and reducing cumulative oxalate burden, underscoring the importance of timely recognition and genetic confirmation.

This review summarizes the genetic and clinical characteristics of the three types of PH, describes strategies for diagnosis, and discusses current as well as emerging therapeutic approaches.

## 2. Epidemiology

The true prevalence of PH is unknown due to frequent underdiagnosis. Estimates suggest a prevalence of fewer than 3 cases per 1,000,000 individuals, though some sources report rates as high as 1 in 58,000. Higher prevalence estimates often derive from carrier-frequency modeling or selected populations, whereas lower estimates typically reflect clinically diagnosed cases, contributing to the wide variability reported in the literature. Type I PH (PH1), the most common form, is estimated to affect 1 to 3 individuals per million population [6,7].

## 3. Genetics

PHs are autosomal recessive disorders caused by pathogenic variants in one of three hepatic enzymes involved in glyoxylate metabolism: alanine–glyoxylate aminotransferase (AGT), glyoxylate reductase/hydroxypyruvate reductase (GRHPR), and 4-hydroxy-2-oxoglutarate aldolase (HOGA1) [8]. These enzymes are encoded by AGXT, GRHPR, and HOGA1, respectively, and mutations in each gene define the three types of PH.

PH1, the most severe and most common form, results from biallelic variants in AGXT, located on chromosome 2q37.3. More than 200 pathogenic or likely pathogenic variants have been described, including missense substitutions, frameshift mutations, splicing abnormalities, and large deletions [9]. Many variants cause misfolding or mistargeting of AGT from the peroxisome to mitochondria, whereas others impair catalytic activity or stability. Certain genotypes correlate with pyridoxine responsiveness, particularly variants such as p.Gly170Arg and p.Phe152Ile, which partially preserve cofactor-dependent folding or activity. PH1 shows wide phenotypic variability, from infantile oxalosis to adult-onset stone disease, partly influenced by genotype [10].

Type 2 PH (PH2) is caused by biallelic variants in GRHPR on chromosome 9p13. GRHPR defects reduce the conversion of glyoxylate to glycolate, diverting glyoxylate toward oxalate formation. Fewer mutations have been reported compared to PH1, and most result in loss of enzyme activity rather than mistargeting [11]. Clinically, PH2 tends to display a milder course than PH1 but can still progress to recurrent nephrolithiasis, nephrocalcinosis, and, in some patients, ESKD.

Type 3 PH (PH3), the least severe form, arises from pathogenic variants in HOGA1 on chromosome 10q24. HOGA1 deficiency disrupts mitochondrial hydroxyproline catabolism, expanding the glyoxylate pool [12]. PH3 typically presents with recurrent stone disease in childhood, and progression to kidney failure is uncommon, though reported.

Given the autosomal recessive inheritance pattern, family screening and genetic counseling are recommended. Genetic testing—via targeted sequencing panels, exome sequencing, or copy-number analysis—is central to confirming the diagnosis, enabling differentiation among PH types, guiding therapy selection (such as pyridoxine trials or RNA interference therapies), and informing prognostic discussions. Table 1 summarizes the genetic and enzymatic defects in PH.

Beyond pyridoxine responsiveness, genotype–phenotype correlations remain imperfect, and substantial interindividual variability exists even among patients carrying identical variants, limiting the predictive value of genotype alone for individual prognosis.

## 4. Oxalate Metabolism and Pathophysiology of PH

Oxalate arises either from endogenous metabolic pathways or from dietary intake through certain foods, beverages, and chemicals. In humans, hepatic production is the dominant source, contributing an estimated 50–80% of total oxalate burden, with glyoxylate serving as the key precursor [13,14].

PH encompasses three distinct forms—types 1, 2, and 3—each caused by a deficiency of a specific hepatic enzyme located in a different intracellular compartment. Regardless of the type, the result is an increased glyoxylate pool and subsequent overproduction of oxalate.

In hepatocytes, glyoxylate is generated from several metabolic substrates, including glycolate, glycine, and hydroxyproline. Under physiological conditions, hepatic glyoxylate metabolism is tightly regulated by a series of mitochondrial, cytosolic, and peroxisomal enzymes that collectively limit its oxidation to oxalate [15].

**Figure 1:**
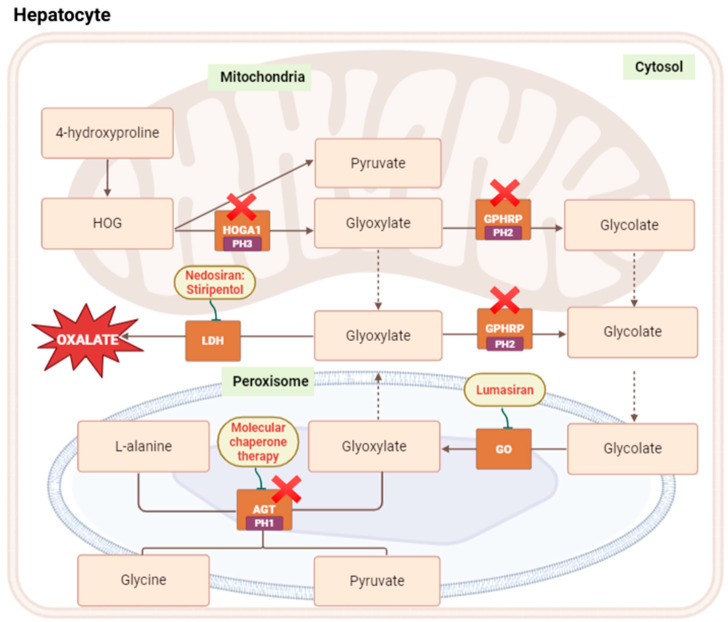
Oxalate metabolism and Pathogenesis of Primary Hyperoxaluria.

Oxalate generation proceeds through three principal steps (Figure 1):

### 4.1. Mitochondrial Hydroxyproline Catabolism

Hydroxyproline, derived largely from collagen turnover and dietary animal protein, is metabolized to 4-hydroxy-2-oxoglutarate (HOG). HOG is subsequently cleaved by 4-hydroxy-2-oxoglutarate aldolase (HOGA1) into glyoxylate and pyruvate [16].

In PH3, deficiency of HOGA1 leads to accumulation of HOG and diversion toward glyoxylate production, expanding the glyoxylate pool and ultimately increasing oxalate synthesis [17].

### 4.2. Conversion of Glyoxylate to Glycolate and Glycolate to Glyoxylate

Glyoxylate originating from mitochondria or from dietary sources can be reduced to glycolate by glyoxylate reductase/hydroxypyruvate reductase (GRHPR) [18]. Glycolate is subsequently transported to the peroxisome, where glycolate oxidase (GO) converts it back to glyoxylate.

Within the peroxisome, alanine–glyoxylate aminotransferase (AGT), a pyridoxal-5′-phosphate–dependent enzyme, detoxifies glyoxylate by transaminating it to glycine and pyruvate [13,19]. This is the central protective step that prevents glyoxylate from being shunted toward oxalate.

### 4.3. Cytosolic Oxidation of Glyoxylate to Oxalate

Any glyoxylate that escapes peroxisomal metabolism diffuses into the cytosol, where it is rapidly oxidized to oxalate by lactate dehydrogenase (LDH).

Defects in the enzymes responsible for these steps underlie the three forms of PH. In PH1, pathogenic variants in AGXT lead to deficiency or mistargeting of AGT, allowing glyoxylate to accumulate. In PH2, loss of function of GRHPR impairs reduction of glyoxylate to glycolate, again favoring its oxidation to oxalate [20]. In PH3, deficiency of HOGA1 increases production of glyoxylate from mitochondrial hydroxyproline metabolism.

Across all types, the common pathway is expansion of the intracellular glyoxylate pool and its subsequent conversion to oxalate, culminating in increased urinary oxalate excretion and CaOx crystal formation.

These enzymatic steps directly inform current therapeutic strategies, including inhibition of glycolate oxidase (lumasiran), suppression of LDH activity (nedosiran and small-molecule inhibitors), and restoration of AGT function through cofactor supplementation or emerging gene-based approaches.

## 5. Clinical Features

The hallmark of PH is excessive endogenous oxalate production, leading to supersaturation of urinary oxalate and progressive deposition of CaOx crystals in renal and extrarenal tissues. Because renal excretion is the primary elimination pathway for oxalate, affected individuals typically exhibit markedly elevated urinary oxalate levels. In most patients, 24 h urinary oxalate excretion exceeds 1 mmol/1.73 m^2^, compared with values below 0.5 mmol/1.73 m^2^ in healthy individuals [21]. The resulting urinary supersaturation promotes intratubular crystal precipitation and recurrent nephrolithiasis, while progressive interstitial deposition contributes to nephrocalcinosis [1,21]. Repeated stone episodes, crystal-induced tubular injury, and recurrent infections ultimately drive chronic kidney disease.

Clinical manifestations usually emerge during early childhood, most often before the age of ten [21]. However, a proportion of patients remain undiagnosed until adulthood, frequently presenting with chronic kidney disease after earlier symptoms were overlooked or attributed to more common causes [22].

PH1 displays substantial phenotypic heterogeneity, even within the same family. It is the most prevalent and most severe form of the disease. Most patients are symptomatic during childhood or early adolescence, commonly with recurrent renal colic and nephrocalcinosis. ESKD typically develops between the third and fifth decade of life, although progression during infancy may occur [21,23]. Infantile oxalosis—a severe presentation seen almost exclusively in PH1—is characterized by ESKD within the first year of life, frequently accompanied by retinal involvement, bone disease, and systemic oxalate deposition [23].

PH2 shares many clinical features with PH1 but tends to follow a less aggressive course, with fewer stone events, lower rates of nephrocalcinosis, and improved renal survival [24].

PH3 is generally associated with the mildest phenotype. Although recurrent nephrolithiasis typically begins in early childhood and urinary oxalate excretion may be comparable to that of PH1 and PH2, overall stone burden and risk of progression are typically lower, and progression to ESKD and systemic oxalosis is rare, with considerable interindividual variability reported across cohorts. [25,26]. Although progression to ESKD is uncommon in PH3, isolated cases have been reported, particularly in patients with early-onset disease, high stone burden, recurrent obstructive episodes, or delayed diagnosis. Long-term follow-up studies and meta-analyses indicate that a small subset of PH3 patients may develop advanced CKD, underscoring the importance of continued surveillance and early metabolic management even in this generally milder phenotype.

In both PH1 and PH2, once significant renal impairment develops—typically at a GFR below 30 mL/min/1.73 m^2^—hepatic oxalate production surpasses renal excretory capacity, resulting in rising plasma oxalate levels. At this stage, systemic oxalosis ensues, with CaOx deposition in multiple organs (Table 2), including the skin, bones, myocardium, and retina [21,27].

Heterozygous carriers of PH-associated variants generally exhibit normal urinary oxalate excretion, maintain preserved kidney function, and experience kidney stones at rates similar to the general population [21].

## 6. Diagnosis

**Figure 2:**
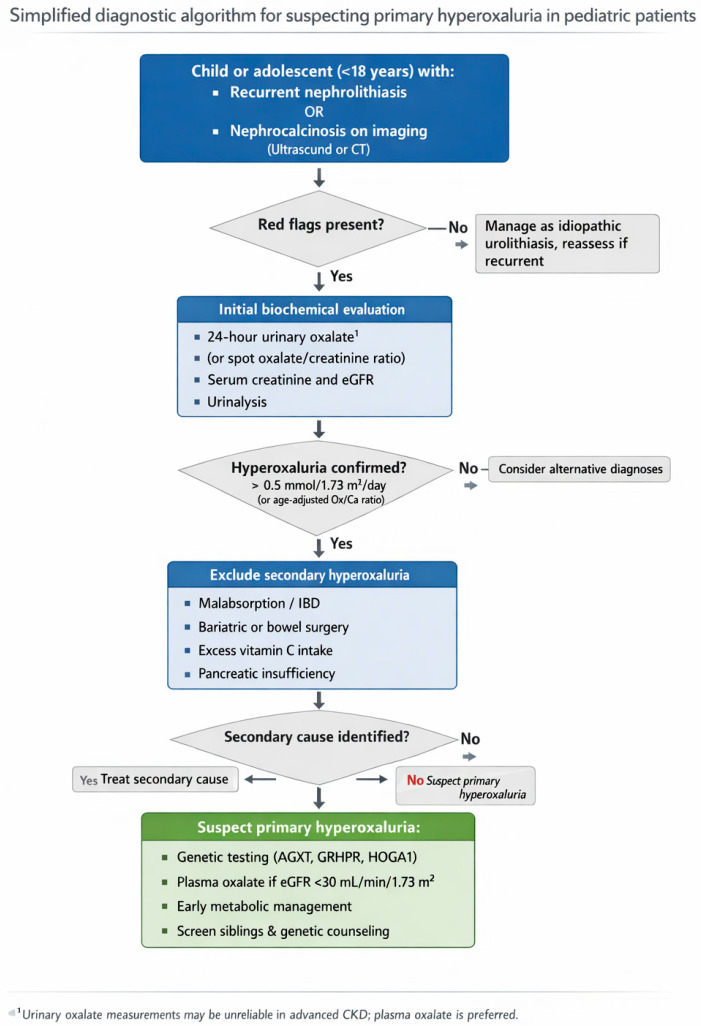
Simplified algorithm for suspecting primary hyperoxaluria in pediatric patients.

PH is frequently underdiagnosed due to its rarity, limited clinical familiarity, and nonspecific early manifestations. It should be suspected in individuals with recurrent nephrolithiasis beginning in childhood or early adolescence, unexplained progressive decline in kidney function, or a family history of kidney stones or kidney disease [28]. To facilitate early recognition in clinical practice, a simplified diagnostic algorithm for suspecting PH in pediatric patients with recurrent nephrolithiasis is presented in Figure 2.

Initial evaluation includes measurement of oxalate excretion in a 24 h urine sample. Hyperoxaluria is confirmed when urinary oxalate exceeds 0.5 mmol/1.73 m^2^ in at least two separate collections, after which secondary causes should be excluded (Table 3) [21]. Urine samples with pH > 8 are not suitable for oxalate analysis, as oxalate can form in vitro under these alkaline conditions [29].

In patients with reduced GFR, 24 h oxalate excretion may be unreliable due to impaired renal clearance. In this setting, the urinary oxalate-to-creatinine ratio (normal < 0.05 mmol/mmol in adults) is preferred [28]. Plasma oxalate measurement, although of limited value when renal function is preserved, becomes essential in CKD stages 4 and 5 for diagnostic confirmation [27,28]. Plasma oxalate levels greater than approximately 50 µmol/L should prompt genetic testing; however, interpretation must consider assay variability and potential overlap with advanced CKD from non-PH etiologies [1].

Additional urinary metabolites from the glyoxylate pathway can support diagnosis. PH1 is often associated with elevated urinary glycolate (seen in up to 75% of cases), though neither its presence nor absence is diagnostic. PH 2 is characterized by increased urinary L-glycerate, while PH3 may show elevated 4-hydroxy-2-oxoglutarate (HOG) and 2,4-dihydroxyglutarate (DHG) [27]. However, detection of these metabolites is limited to specialized centers and may not be widely accessible.

Once biochemical findings suggest PH, genetic testing is strongly recommended, as identification of the causative mutation confirms the diagnosis, informs prognosis, and guides therapeutic decision-making. With the increasing availability of comprehensive genetic testing, liver biopsy is now rarely required and should be reserved for exceptional cases with strong clinical suspicion and inconclusive genetic results, to assess AGT, GRHPR, or HOGA enzyme activity in hepatocytes [1].

**Figure 3:**
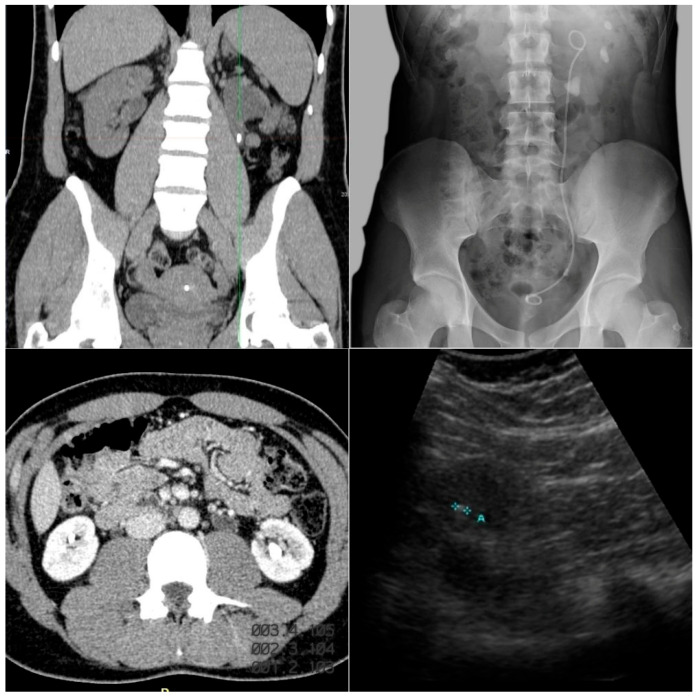
Representative imaging of nephrolithiasis in our case series of patients with primary hyperoxaluria in Hospital Universitario La Paz. Computed tomography (**left images**): coronal section (**top**) and axial section (**bottom**) showing hyperdense foci within the urinary system, consistent with urolithiasis. Plain abdominal radiograph (**top right**): a double J ureteral stent is identified on the left side, associated with radiopaque calcified densities projected onto the renal silhouette, consistent with lithiasis. Ultrasound (**bottom right**): hyperechoic focus with posterior acoustic shadowing, a characteristic finding of urinary lithiasis.

Imaging studies should be performed to detect nephrolithiasis or nephrocalcinosis [24]. Nephrocalcinosis is especially relevant in PH because it reflects higher systemic oxalate load and is associated with a worse renal prognosis [30]. In these patients, CaOx deposition generates diffuse medullary calcifications, often described as “bright papillae” or an “arborescent pattern”, with severity correlating directly with kidney function decline [30,31] (Figure 3).

The main imaging techniques include:

*Ultrasound:*

First-line modality due to its safety (no radiation), reproducibility, and low cost. It is particularly useful in children. It identifies calculi in calyces or renal pelvis and detects medullary hyperechogenicity and corticomedullary differentiation loss—typical signs of nephrocalcinosis [31].

*Plain abdominal X-ray:*

Serves as a complementary tool in stable patients, often combined with ultrasound. It detects radiopaque stones, and CaOx calculi are commonly visible [32].

*Non-contrast CT:*

The gold standard for urinary stone evaluation, with higher sensitivity than ultrasound or intravenous urography. It precisely localizes and characterizes stones, estimates density and composition via Hounsfield Units, and assesses parenchymal calcifications such as nephrocalcinosis [33,34].

Following confirmation of PH, family screening is advised, as early detection of affected siblings—who may be asymptomatic yet have significant oxalate burden—allows timely intervention and prevention of disease progression [24].

## 7. Current Treatment

### 7.1. Medical Treatment

Medical treatment in PH aims to reduce urinary supersaturation of CaOx, prevent crystal formation, delay kidney damage, and limit systemic oxalate deposition. Management must be individualized according to PH subtype, kidney function, and responsiveness to pyridoxine or RNA-interference (RNAi) therapies.

#### 7.1.1. Conservative Measures

##### Hyperhydration

Urine dilution remains the cornerstone of conservative therapy [35]. Although diuresis > 1 mL/kg/h nearly eliminates CaOx supersaturation in non-PH stone formers, patients with PH require substantially higher volumes. Current recommendations target a daily fluid intake of 3.5–4 L in adults and ≥2–3 L/m^2^ BSA in children, aiming for ≥2.5 L of urine/day [36]. Infants may require gastrostomy-assisted hydration.

Monitoring includes morning spot urine oxalate/creatinine ratios and, when feasible, crystalluria assessment to titrate hydration intensity [37].

##### Citrate Supplementation

Potassium citrate (0.1–0.15 g/kg) may reduce CaOx crystallization through calcium binding and urinary alkalinization, with an aim of increasing urine pH > 6.5, although data are limited and inconsistent [38,39]. Given its mechanistic rationale, citrate is recommended as part of the standard therapeutic regimen, with monitoring of urinary pH and tolerance.

##### Dietary Measures

Evidence on dietary oxalate restriction is conflicting [40,41]. Diet contributes variably to urinary oxalate, with absorption influenced by calcium intake, oxalate bioavailability, and the intestinal microbiome. Given the minimal and variable impact on urinary oxalate and the negative effect on quality of life, strict low-oxalate diets are not recommended [27]. Instead, patients should avoid only foods with extremely high oxalate content, such as spinach, rhubarb, chocolate, and nuts. Controlled diet studies indicate that urinary oxalate rises approximately 1.7 mg for every 100 mg of dietary oxalate ingested when calcium intake is maintained at 1000 mg/day [42]. Increasing dietary calcium reduces intestinal oxalate absorption by forming insoluble complexes, highlighting the importance of coordinated dietary management.

#### 7.1.2. Pyridoxine Therapy

Pyridoxine (vitamin B6) lowers oxalate production in a subset of patients with PH1, particularly those carrying non-truncating AGXT variants (e.g., p.Gly170Arg, p.Phe125Ile, p.Gly41Arg) [43,44,45,46].

##### Indications and Dosing of Pyridoxine Therapy

Pyridoxine should be initiated in:

- all patients suspected of PH1, pending genetic confirmation;
- all patients with genetically confirmed PH1.

The maximum recommended dose is 5 mg/kg/day, as higher doses show no proven benefit and carry risks of neurotoxicity [27].

##### Assessment of Responsiveness

Responsiveness is defined as a >30% reduction in urinary oxalate after ≥2 weeks (short-term) or ≥3 months (full evaluation) of therapy, confirmed on two separate measurements. Responsive patients should undergo dose tapering to the minimum amount maintaining the oxalate reduction, guided by repeated urine oxalate testing [21].

Non-responders do not require frequent monitoring unless receiving RNAi therapy. In responders, urinary oxalate should be monitored closely until an effective dose is established, and subsequently twice per year [27].

#### 7.1.3. RNA-Interference (RNAi) Therapies

Until recently, management of PH1 relied mainly on supportive measures that were demanding for patients and only partially effective. Even when hyperhydration and citrate therapy are followed rigorously, they do not reliably prevent progression to ESKD. New treatments—particularly RNA interference–based (RNAi) therapies—have shown encouraging short-term results in lowering oxalate production. Growing clinical evidence suggests that these agents may significantly transform the treatment landscape for PH1 in the coming years [47,48]. Long-term effects remain under investigation, but observational cohorts (30–48-month follow-up) indicate sustained biochemical and clinical benefit.

Most available evidence for RNA interference therapies is based on biochemical surrogate endpoints, including reductions in urinary and plasma oxalate, while robust long-term clinical outcome data remain limited. Comparative efficacy between agents should be interpreted cautiously, as no head-to-head trials exist. Evidence strength is strongest for PH1, inconclusive or negative for PH2, and currently limited for PH3. Long-term metabolic, hepatic, and neurodevelopmental safety—particularly in pediatric patients receiving lifelong therapy—requires continued surveillance through prospective registries and long-term follow-up studies.

##### Lumasiran

Lumasiran (approved by EMA and FDA for the treatment of PH1) is a synthetic double-stranded RNA interference agent that reduces oxalate production by targeting the mRNA for glycolate oxidase, thus inhibiting glycolate oxidase, reducing glyoxylate and subsequently oxalate production. In the ILLUMINATE trials, Lumasiran has shown a ~65–72% reduction in urinary oxalate in PH1; and 50–84% of patients reach oxalate < 1.5× upper reference limit, with a sustained biochemical response up to at least 12 months [49,50]. Efficacy has been demonstrated across adults, children, and infants; with very good tolerability and mild adverse effects (mainly injection-site reactions). In patients with reduced eGFR or on dialysis, Lumasiran lowers plasma oxalate by ~30–40% [51].

##### Nedosiran

Nedosiran is a synthetic double-stranded RNA interference agent that targets the mRNA for lactate dehydrogenase isoform (LDHa), and theoretically applies to all PH types. The PHYOX clinical trials have shown a ≈55–60% urinary oxalate reduction in PH1 [52,53,54], maintaining this effect for at least up to 42 months. In PHYOX3, normalization or near-normalization of 24 h urinary oxalate excretion was achieved for most participants from month 2 onward [54], and in PHYOX8, Nedosiran treatment led to normalization or near normalization of urine oxalate levels in 93% of children with PH1 [55]. Early case reports suggest possible benefit even in dialysis-dependent PH1 [56]. Nedosiran has shown no significant benefit in PH2 [53], likely due to the systemic distribution of LDH isoenzymes and the contribution of extrahepatic oxalate production. While hepatic LDHA inhibition effectively reduces oxalate generation in PH1, PH2 involves impaired cytosolic glyoxylate metabolism, and residual oxalate production in extrahepatic tissues may attenuate the biochemical response to liver-targeted RNA interference. Early phase data suggest potential efficacy of nedosiran in PH3. In the PHYOX4 phase 1 study, patients with PH3 demonstrated an average urinary oxalate reduction of approximately 24% by day 85, with some individuals achieving reductions greater than 30% [57]. Although these data are preliminary and derived from small cohorts, they provide initial evidence that hepatic LDHA inhibition may also benefit selected PH3 patients.

A recent meta-analysis confirmed a significant reduction in urinary and plasma oxalate with RNAi inhibitors, along with a favorable overall safety profile. Lumasiran showed greater efficacy than nedosiran, along with a clear dose–response effect and sustained long-term benefits. Furthermore, the therapeutic effect was even greater in patients undergoing hemodialysis [58].

#### 7.1.4. Dialysis

##### Indications of Dialysis in PH

Dialysis is considered in CKD stage 4–5 when plasma oxalate is elevated despite oxalate-lowering treatments or when signs of systemic oxalosis are present. Because plasma oxalate accumulation reflects ongoing tissue deposition, early dialysis may be needed before overt uraemia.

##### Limitations of Conventional Dialysis and Recommended Strategies

Endogenous oxalate production in PH1 (4–7 mmol/day) [59] far exceeds removal by standard dialysis (≈1–1.4 mmol/day) [60], making conventional regimens insufficient to prevent systemic accumulation. Therefore, intensive haemodialysis (HD) with high-flux membranes and maximal blood flow is preferred, with daily sessions when tolerated [61,62]. Peritoneal dialysis (PD) is not recommended, as higher oxalate clearance per minute is achieved with HD (≈116 mL/min/1.73 m^2^) compared with PD (≈7 mL/min/1.73 m^2^) [60].

Session frequency should be increased rather than duration, as removal decreases over the course of each session [61]. Pre-HD plasma oxalate levels of approximately 50–70 μmol/L should be targeted, similar to values in non-PH dialysis patients, with consideration of residual diuresis and inter-laboratory variability [27,63]. In patients receiving intensive frequent HD, phosphate control is essential to avoid worsening of bone disease in patients receiving intensive dialysis. Decisions regarding daily dialysis should involve shared decision-making, accounting for patient tolerance and caregiver burden.

#### 7.1.5. Transplantation

##### Liver Transplantation

For PH1, liver transplantation is the only definitive cure, correcting the metabolic defect and halting oxalate overproduction. Auxiliary liver transplantation is not recommended due to inadequate metabolic correction [64].

##### Kidney Versus Combined Liver–Kidney Transplantation (CLKT)

In patients with PH1 and advanced kidney failure, CLKT provides superior kidney graft survival compared with isolated kidney transplantation (87% vs. 14% at 15 years) [65]. CLKT also improves event-free survival in pyridoxine-non-responsive patients [66]. In pyridoxine-responsive PH1 with normalization or near normalization of urinary oxalate excretion, outcomes of isolated kidney transplantation may be acceptable, allowing a selective approach [27]. Metry et al. showed that simultaneous and sequential CLKT have comparable outcomes [66]. In this study, pre-emptive liver transplantation without kidney involvement showed poor results and is not advised.

Most transplantation outcome data derive from the pre-RNAi era and may not fully reflect future clinical practice. Effective oxalate-lowering therapies may modify transplant timing and strategy, potentially allowing postponement of combined liver–kidney transplantation or consideration of isolated kidney transplantation in carefully selected responsive patients, particularly in pediatric populations.

##### Transplantation in PH2

Data are limited, but isolated kidney transplantation has shown poor graft survival in PH2. While successful CLKT cases exist, results are mixed, and indications must be individualized [49].

### 7.2. Surgical Treatment

Surgical management in PH aims to:

1. Remove symptomatic calculi causing pain, infection, obstruction, or renal deterioration;
2. Restore urinary drainage in cases of acute obstruction;
3. Minimize renal parenchymal injury and reduce recurrence by prioritizing minimally invasive techniques; and
4. Prevent progression toward ESKD. Because patients with PH have a high stone-recurrence burden and are at risk of systemic oxalosis, surgical decisions must be individualized and coordinated within a multidisciplinary team [8,9].

#### 7.2.1. Indications for Urgent Urinary Drainage

Identifying acute obstructive uropathy is essential, as it requires immediate decompression prior to definitive stone treatment. Although no PH-specific protocols exist, indications parallel those for other forms of obstructive urolithiasis. Table 4 summarizes the radiological features suggestive of acute obstructive uropathy. Emergency intervention is required in the presence of: (1) radiologically confirmed acute obstruction; (2) associated urinary infection or sepsis; (3) severe, refractory renal colic; (4) rapidly worsening kidney function (rising creatinine, sudden eGFR decline); or (5) high stone burden in the context of marked hyperoxaluria (>0.7 mmol/1.73 m^2^/24 h) [33,67]. Patients with advanced CKD (eGFR < 30–40 mL/min/1.73 m^2^) or at risk of systemic oxalosis should also be managed urgently, with drainage and, if needed, initiation of dialysis [68].

Two decompression techniques are used; percutaneous nephrostomy (PCN) and double-J ureteral stents (DJ). The choice between PCN and DJ depends on clinical context, anatomical considerations, operator experience, and resource availability [68,69].

*Percutaneous nephrostomy (PCN)*

Placement of a drainage catheter directly into the renal pelvis under ultrasound or fluoroscopic guidance. PCN is preferred in severe sepsis, complete obstruction, anuria, anatomical abnormalities, or when retrograde access is not feasible [68,69].

*Double-J ureteral stent*

Inserted retrogradely via cystoscopy to re-establish drainage from kidney to bladder. It is favored in ureteral obstruction without severe sepsis and when anatomy and anesthesia risk are suitable [68,69].

#### 7.2.2. Definitive Stone-Treatment Techniques

Stone-removal strategies in PH must aim to minimize the number of procedures and avoid renal injury, as these patients have high recurrence rates and a significant risk of CKD progression [27,31,33,67]. Technique selection depends on stone size, composition, anatomical factors, and overall renal function.

*Extracorporeal Shock Wave Lithotripsy (ESWL)*

ESWL delivers acoustic shock waves externally to fragment renal or ureteral stones, usually on an outpatient basis under local anesthesia or mild sedation [70].

However, CaOx monohydrate stones—commonly seen in PH—are dense, hard, and relatively resistant to ESWL, particularly when Hounsfield units exceed 1000 or when stones are located in the lower pole. Moreover, ESWL carries increased risk of renal injury in children, patients with nephrocalcinosis, and individuals with impaired kidney function [9,33]. For these reasons, ESWL is generally considered a secondary option in PH.

*Ureteroscopy (URS)*

URS is the preferred minimally invasive approach for most PH patients. Flexible or semirigid ureteroscopes allow direct visualization, intracorporeal laser lithotripsy, and extraction of fragments. URS is effective for ureteral and intrarenal stones, associated with low morbidity, and is well suited to patients requiring repeated interventions [70,71]. Post-operative stent placement is individualized.

*Percutaneous Nephrolithotomy (PCNL)*

PCNL provides percutaneous access to the collecting system to fragment and extract large or complex stones. It is the treatment of choice for stones > 20 mm, staghorn calculi, or multiple stones not amenable to URS [70,71].

While PCNL offers high stone-free rates, it carries risks such as bleeding, infection, and post-operative acute kidney injury—particularly relevant in PH with fragile renal parenchyma. Nonetheless, in high stone-burden scenarios it remains indispensable.

Overall, technique selection should be individualized, prioritizing renal preservation and minimizing repeated interventions.

#### 7.2.3. Stone Composition Analysis

Stone analysis is essential for all PH patients as it guides metabolic evaluation, medical therapy, and recurrence prevention [27]. PH typically produces CaOx stones, and the two major crystalline forms have different clinical implications:

*Calcium Oxalate Monohydrate (Whewellite)*

- Dense, compact, and highly resistant to fragmentation by ESWL.
- Common in PH and other severe metabolic disorders.
- Radiopaque on imaging with high Hounsfield density [32,34].

*Calcium oxalate dihydrate (weddellite)*

- Less dense, more fragile, and more amenable to ESWL.
- More typical of idiopathic stone disease or milder metabolic derangements [32,34].
- Mixed stones containing calcium phosphate or uric acid may indicate co-existing metabolic abnormalities and require tailored preventive strategies.

#### 7.2.4. Metabolic Monitoring, Post-Treatment Surveillance and Residual Fragments

PH represents a high-risk condition for recurrent stone formation and systemic complications. Thus, targeted prophylaxis and structured follow-up are essential.

After initiating therapy, a follow-up metabolic evaluation is recommended at 8–12 weeks to adjust treatment. Once urinary parameters stabilize, at least a biannual 24 h urine testing is required for monitoring PH1.

Residual fragments markedly increase recurrence risk. Routine follow-up typically includes ultrasound or plain radiography; CT is reserved for symptomatic patients or pre-intervention planning to limit cumulative radiation exposure. Evaluation should occur approximately four weeks after definitive treatment to avoid misinterpretation of transient debris [72].

## 8. Pharmacologic Innovations and Emerging Treatments

Despite advances in pharmacological and RNAi-based therapies for PH, significant challenges remain in fully preventing oxalate accumulation, kidney stones, and progressive renal damage. Emerging research is expanding beyond enzyme cofactor supplementation and RNAi, focusing on complementary strategies, such as targeting intestinal oxalate absorption, modulating the gut microbiome, deploying oxalate-degrading enzymes, and gene editing, which may provide new avenues to reduce oxalate burden and prevent kidney damage. While many strategies are still in early clinical or preclinical stages, their integration with established approaches could enable more comprehensive, personalized management of PH in the coming years.

### 8.1. Microbiome-Based Approaches

Certain gut bacteria, particularly *Oxalobacter formigenes*, metabolize oxalate as their sole carbon source and may help reduce systemic oxalate burden. *O. formigenes* also appears to secrete a factor that enhances intestinal oxalate secretion via the anion transporter SLC26A6 [73]. Early animal studies suggested that intestinal colonization could lower plasma oxalate [74], and a case report in an 8-week-old PH1 infant with nephrocalcinosis and anuric kidney failure showed a decrease in plasma oxalate from 130 to 80 μmol/L after five months of oral O. formigenes administration [75].

Clinical data remain mixed but encouraging. In a phase II trial, 12 PH1 patients with kidney failure received an oral formulation for six weeks, followed by washout. Although short-term effects were inconclusive, eight patients continued in a 36-month extension study, where plasma oxalate levels declined from 147 to 120 μmol/L at 12 months and to 95 μmol/L at 24 months. In contrast, a contemporaneous natural-history cohort on dialysis showed no spontaneous oxalate decline [76]. A recent phase III trial of a lyophilized formulation suggested stabilization or reduction of plasma oxalate compared with placebo, though differences were not statistically significant [77]. Modifying the microbiome thus remains a promising, non-invasive adjunct to current therapies.

### 8.2. Stiripentol

Stiripentol, an antiepileptic drug that inhibits lactate dehydrogenase, has been explored as a potential therapy to reduce hepatic oxalate production in PH1. Preclinical studies showed decreased urinary oxalate and renal deposition in hyperoxaluric rats, and several case reports—as well as observations in children with Dravet syndrome—demonstrated substantial reductions in urinary oxalate [78,79]. However, other reports, particularly in patients with advanced CKD, found no clinical benefit [80,81]. A phase 2 trial evaluating its ability to lower urinary oxalate was completed in March 2021 (NCT03819647), and overall efficacy remains uncertain.

### 8.3. Lanthanum

Lanthanum carbonate, a phosphate binder with minimal systemic absorption, can also bind intestinal oxalate. In animal models it reduced urinary oxalate, crystalluria, and nephrocalcinosis [82]. Small clinical studies in PH1—including a proof-of-concept trial and two case reports—showed marked reductions in plasma and urinary oxalate, even in advanced CKD [83,84]. Although early findings are encouraging, larger studies are needed to confirm efficacy and safety.

### 8.4. Oxalate-Degrading Enzymes

Exogenous enzymes capable of degrading intestinal oxalate are in development. Oxalate decarboxylase-based formulations, such as Oxazyme and Reloxaliase, aim to reduce oxalate absorption in the gut before it reaches the urinary tract. Early studies in enteric hyperoxaluria show reductions in urinary oxalate, and these approaches may be extended to PH or idiopathic CaOx stone formers [85,86]. Enzyme therapy offers a potentially adjunctive, non-systemic intervention with minimal systemic side effects.

### 8.5. Gene Editing and Other Novel Strategies

Gene-editing strategies such as CRISPR/Cas9 delivered via adeno-associated viral vectors are emerging as potentially durable treatments for primary hyperoxaluria. By directing the Cas9 endonuclease to specific hepatic genes involved in oxalate production, this approach can induce targeted insertions or deletions that disrupt enzyme activity. In PH1 animal models, CRISPR/Cas9 knockout of LDHA achieved loss of gene function in roughly 20% of hepatocytes, lowering LDH expression by half and significantly reducing urinary oxalate and renal calcium oxalate deposition without detectable hepatotoxicity or off-target effects [87]. Targeting HAO1 similarly produced substantial reductions in urinary oxalate and prevented nephrocalcinosis over long-term follow-up [88,89]. Despite promising preclinical results, gene-editing approaches face substantial uncertainties regarding long-term durability due to incomplete hepatocyte targeting with the potential need for retreatment as the liver regenerates, as well as other issues such as off-target effects and lifelong safety. Careful clinical evaluation and prolonged surveillance will be essential before these strategies can be considered for routine clinical use. Although these results are promising. A gene editing strategy targeting the glycolate oxidase gene in humans is under investigation (NCT06839235).

Alongside these genome-editing approaches, small-molecule inhibitors are under investigation, which may eventually complement or even supersede current therapies. Findings from animal studies evaluating an orally delivered, liver-targeted LDH inhibitor and AGT mRNA replacement therapy suggest these approaches could become valuable future strategies for managing PH [90,91].

## 9. Conclusions and Future Directions

Despite substantial advances in understanding the underlying genetic and enzymatic defects in PH, it remains frequently underdiagnosed, often only identified after significant renal injury has occurred. Early recognition—supported by biochemical testing, genetic confirmation, and appropriate imaging—remains critical to prevent irreversible kidney damage and systemic oxalosis.

Current management requires a multidisciplinary approach that integrates conservative measures, pyridoxine therapy for responsive PH1 genotypes, and RNA interference therapies that directly modulate hepatic glyoxylate metabolism and have reshaped the therapeutic landscape. Within this framework, surgical and endourological interventions—such as ureteroscopy, percutaneous nephrolithotomy, and, when appropriate, minimally invasive stone removal—remain important for managing obstructive episodes, preserving renal function, and addressing the substantial stone burden. For advanced disease, intensive dialysis strategies and timely consideration of combined liver–kidney transplantation remain essential. Nevertheless, existing treatments do not fully prevent oxalate accumulation or disease progression in all patients.

Future directions encompass strategies targeting intestinal oxalate handling, modulation of the gut microbiome, and development of orally administered oxalate-degrading enzymes. Emerging gene-editing technologies, including CRISPR-based approaches and hepatocyte-directed mRNA or small-molecule therapies, hold potential for durable metabolic correction, though long-term safety and efficacy must be established. Continued translational research, earlier diagnostic pathways, and individualized therapeutic algorithms will be central to improving outcomes and reducing the global burden of PH.

## Figures and Tables

**Table 1:** Genetic and Enzymatic Defects in Primary Hyperoxaluria.

PH Type	Gene	Encoded Enzyme	Cellular Localization	Pathogenic Mechanism	Typical Clinical Severity
PH1	AGXT	Alanine–glyoxylate aminotransferase (AGT)	Peroxisome	Enzyme deficiency, misfolding, or mistargeting of AGT → accumulation of glyoxylate → increased oxalate	Most severe; early nephrolithiasis, nephrocalcinosis, risk of ESKD and systemic oxalosis
PH2	GRHPR	Glyoxylate reductase/hydroxypyruvate reductase	Cytosol	Reduced conversion of glyoxylate to glycolate → enhanced glyoxylate oxidation to oxalate	Intermediate severity; recurrent stones, variable risk of progression to ESKD
PH3	*HOGA1*	4-hydroxy-2-oxoglutarate aldolase (HOGA1)	Mitochondria	Impaired hydroxyproline catabolism → increased glyoxylate synthesis	Least severe; recurrent stones in childhood, rare progression to ESKD

**Table 2:** Extrarenal Manifestations of Primary Hyperoxaluria.

System/Organ	Manifestations
Skin	Livedo Reticularis-like rash, subcutaneous nodules and acrocyanosis
Heart	Cardiomyopathy, conduction disorders
Bones	Malformations, osteolytic lesions and fractures
Bone Marrow	Pancytopenia and erythropoietin-resistant anemia
Nervous System	Peripheral neuropathy
Retina	Visual disturbances

**Table 3:** Causes of Secondary Hyperoxaluria.

Causes
Inflammatory Bowel Disease Primary Biliary Cirrhosis
Short Bowel Syndrome; Partial gastrectomy, small bowel resection
Exocrine Pancreatic Insufficiency
Bariatric Surgery
Medications: orlistat, high vitamin C intake, octreotide, mycophenolate
Ethylene glycol poisoning

**Table 4:** Radiological findings suggestive of acute urinary tract obstruction.

**Hydronephrosis**	Dilation of the renal pelvis and calyces, visible on ultrasound, computed tomography (CT), or intravenous urography (IVU); the most sensitive and specific finding for acute obstruction.
**Hydroureter**	Dilation of the ureter proximal to the obstruction site, particularly evident on CT, IVU, and ultrasound.
**Delayed contrast excretion**	In contrast-enhanced studies or excretory phase imaging (CT urography or IVU), delayed or absent renal excretion indicates significant obstruction.
**Perirenal edema or fluid**	Presence of fluid in the perirenal space, detectable on CT or ultrasound, associated with acute obstruction.
**Persistent or dense nephrogram**	Seen on contrast studies, indicating complete or severe obstruction.
**Urine extravasation**	Less common but serious finding, indicating urinary tract rupture due to obstruction and high pressure.

## Data Availability

No new data were created or analyzed in this study.

## Abbreviations

The following abbreviations are used in this manuscript:

AGT	Alanine–glyoxylate aminotransferase
CaOx	Calcium Oxalate
CLKT	Combined Liver-Kidney Transplantation
CT	Computerized tomography
ESKD	End-stage kidney disease
ESWL	Extracorporeal Shock Wave Lithotripsy
GFR	Glomerular Filtration Rate
GRHPR	Glyoxylate reductase/hydroxypyruvate reductase
HD	Hemodialysis
HOG	4-hydroxy-2-oxoglutarate
HOGA1	4-hydroxy-2-oxoglutarate aldolase
IVU	Intravenous urography
LDH	Lactate dehydrogenase
PCN	Percutaneous nephrostomy
PCNL	Percutaneous nephrolithotomy
PH	Primary Hyperoxaluria
PH1	Type 1 Primary Hyperoxaluria
PH2	Type 2 Primary Hyperoxaluria
PH3	Type 3 Primary Hyperoxaluria
RNAi	RNA interference
URS	Ureteroscopy
